# Using a Plasma Focused Ion Beam System to Characterise the Porosity Through the Oxide Scale Formed on a Martensitic 9Cr-1Mo Steel Exposed to CO_2_

**DOI:** 10.1007/s11085-026-10394-2

**Published:** 2026-05-11

**Authors:** Lawrence Coghlan, Aya Shin, Jonathan Pearson, Mark A. E. Jepson, Rebecca L. Higginson

**Affiliations:** 1 University of Bristol, Physics, Tyndall Ave, Bristol, BS8 1TL UK; 2 Department of Materials, Loughborough University, Epinal Way, Loughborough, LE11 3TU UK; 3 EDF Energy, Javelin House, Building 1420, Charlton Court, Gloucester Buisness Park, Gloucester GL3 4AE UK

**Keywords:** Steel, FIB, SEM, Oxidation, Carburisation, Reconstruction

## Abstract

Focused ion beam (FIB) microscopy, in combination with scanning electron microscopy (SEM) has been used to characterise the porosity through the oxide scale of an experimental martensitic 9Cr-1Mo steel exposed for 3325 h to a CO_2_-rich environment at 600 °C. A typical magnetite outer layer forms with a small fraction of spherical pores, but no interconnectivity between them. A complex mixed middle layer is observed which consists of larger magnetite grains, smaller spinel grains and some pore interconnectivity, but typically the porosity is present in localised regions, inhibited from coalescence by the different grain types. A spinel layer also shows greater pore interconnectivity with three large pores spanning the total distance through this layer, but no single pore spans the total oxide scale thickness. A mechanism linking the substrate microstructure to the variation in oxide structure between ferritic and martensitic material is proposed which explains the formation of the magnetite, the complex oxide and spinel oxide layers.

## Introduction

9Cr-1Mo steel is widely used within the energy generation industry due to its chemical and mechanical properties, high creep strength and sufficient corrosion resistance at medium to high temperatures to allow use for extended lengths of time. One specific use of 9Cr-1Mo steel is within advanced gas-cooled reactors, where components can be exposed to high-temperature CO_2_. To inhibit corrosion in these environments, 9Cr-1Mo steel typically develops a duplex oxide scale which consists of an outer Fe-rich magnetite layer and an inner Cr-rich oxide spinel which provides the corrosion resistance of the alloy [1–4]. Both oxide layers show different morphologies with a clearly identifiable interface.

When exposed to a CO_2_-rich atmosphere, this duplex oxide grows via the available space model [5–11], with the Fe from the substrate diffusing outwards leading to the growth of the outer magnetite (Fe_3_O_4_) oxide layer. O_2_ diffuses inwards towards the substrate, leading to the formation of the Cr-rich, Fe-deficient inner spinel oxide layer growing inwards. As the oxide continues to grow, the diffusion distance increases, with the total oxide thickness and the ratio between the magnetite and Cr-rich spinel changing from ~ 1:1 magnetite: Cr-rich spinel to 1: > 1 ratio [8]. This change away from the 1:1 ratio is not predicted by the available space model and hence is linked to the initiation of breakaway with the spinel layer growing at a greater rate than previously.

“Breakaway” describes the point at which the oxide moves from a protective growth regime to a highly accelerated rate leading to failure of the scale. Various factors can affect time to breakaway, but an overriding initiation point has not been fully agreed. However, under these conditions, Cr being retained in carbides and geometry are considered major factors [10, 12–19]. One factor linked with breakaway oxidation is the carbon and carbide concentration within the substrate which, during exposure to a CO_2_-rich atmosphere, increases with exposure time. If breakaway oxidation occurs, the rate of oxidation shows an exponential increase as the substrate is exposed to the aggressive environmental conditions. The porosity within the oxide scale is important to consider because any pores present within the oxide offer a reduced diffusion resistance through the oxide in comparison to a non-porous oxide. The presence of a complete pore network spanning through the total oxide would affect the oxidation characteristics of the steel and may lead to more rapid initiation of breakaway oxidation.

Experimental 9Cr-1Mo steel samples exposed to CO_2_-rich environments have been shown to form an increased volume of carbides in the near-surface substrate relative to samples exposed to steam or air [9, 13, 20, 21]. This is due to the complex interactions which are taking place between gas, metal substrate and the oxide. The reaction behind these, and carbon formation, is the Boudouard reaction, which is detailed in Eq. 1 and 2. These reactions dictate the form of species which are present within the oxide scale.

1 $${\mathbf{2CO}}_{{\mathbf{2}}} \mathop \rightarrow {\mathbf{2CO}} + {\mathbf{O}}_{{\mathbf{2}}}$$

2 $${\mathbf{2CO}} \to {\mathbf{CO}}_{{\mathbf{2}}} + {\mathbf{C}}$$

During exposure, the steel substrate is oxidised by the CO_2_ which is reduced to CO. This leads to a local increase in CO concentration and the formation of C (Eq. 2). C can be absorbed by the metal leading to the formation of metal carbides within it. The driving force of these reactions is the stability of the carbides and the reduced O_2_ concentration at the oxide/metal interface [11, 14, 22–24]. These carbides increase in size and volume with exposure time, initially increasing in frequency but then decreasing at later stages due to carbide coalescence [3, 24–27]. The formation and subsequent development of these carbides are affected by the microstructure of the substrate [25] and will result in local chemical composition variations as they form. These elemental changes within the carburised layer will lead to uneven elemental distributions within the Cr-rich spinel [25, 26] which are linked with the morphology of the oxide scale. The previous work has proposed that the Cr-rich oxide areas in this spinel layer inhibit pore migration and coalescence [2, 27]. This pore formation is generally attributed to be a result of reduced Fe diffusion outwards, the volume of which will continue to reduce as the oxide thickness increases [19] due to the continued increase in total oxide thickness inhibiting diffusion.

During exposure of the steel to CO_2_, pore development takes place across the oxide, with different morphologies forming across the oxide scale [2], with the oxide and pore features and morphologies being linked with the original substrate. The pores formed in the different oxide layers are distinctly different, with the magnetite layer showing a higher frequency of individual pores and the spinel oxide layer showing greater interconnectivity, with the total interconnectivity changing through the oxide scale. The previous work [2, 27, 28] has investigated the porosity development in 9Cr-1Mo steel and found that there is no evidence of a complete network spanning the total oxide scale thickness. The presence of such a pore network within the oxide scale has the potential to drastically affect the oxidation characteristics of the steel.

The substrate microstructure affects the oxidation characteristics of the material, with martensitic and ferritic steels showing different oxide microstructures [25]. This previous work was performed in 2D (SEM top-down characterisation) and focused on how the prior substrate microstructure affects the carbide development, and subsequent oxide formation. Porosity of a ferritic material has previously been characterised in 3D through an oxide formed on an equiaxed ferritic 9Cr-1Mo steel exposed to a CO_2_-rich atmosphere [2]. The work presented here builds on both previous studies and compares the oxide development on a martensitic substrate in 3D to that of the equiaxed ferritic steel previously reported.

## Experimental Procedures

Experimental 9Cr-1Mo steel samples have been exposed to a CO_2_-rich atmosphere for varying lengths of time on behalf of EDF Energy, with the aim to better understand the corrosion of 9Cr-1Mo steel. The elemental composition of the as-received materials for both DNB (martensitic) and HRA (ferritic) prior to exposure can be seen in Table 1. Both materials were supplied by EDF Energy with heat treatment for both as AGR representative materials.

**Table 1:** Elemental composition of the as received DNB and HRA material (wt%)

Element	C	Cr	Mo	Mn	P	Ni	Co	Cu	Nb	Ti	V	W	Si
As received DNB	0.083	8.6	0.99	0.43	0.01	0.21	0.02	0.09	< 0.02	< 0.02	< 0.05	< 0.05	0.43
As received HRA	0.093	9.2	1.04	0.47	0.011	0.21	0.02	0.16	< 0.02	< 0.02	< 0.05	< 0.05	0.67

These values were measured by Inductively Coupled Plasma Optical Emission Spectroscopy (ICPOES) and were supplied by EDF Energy from their on-going experimental program

The details of the exposures are given in Table 2. Samples were exposed in autoclaves with an inner volume of 22.5 L at a pressure of 4.24 MPa (600 psig) and a gas composition consisting of 0.01% H_2_, 0.03% CH_4_, 0.07% H_2_O, 1% CO by volume with the remainder of the volume being made up of CO_2_. Gas continuously flowed through at a rate of 20 cc/min (at standard temperature and pressure). These conditions are highly carburising and will lead to carburisation of the substrate.

**Table 2:** Sample ID and exposure conditions of samples

Sample reference	Exposure time (h)	Breakaway oxidation?
Unexposed DNB/HRA	N/A	N
DNB-8401-3325	3325	N
DNB-8418-5845	5845	Y
HRA-4580	4580	N

Upon removal from the autoclaves, the samples were labelled as having suffered breakaway, or no breakaway. This was done visually by comparison with previous work on similar samples. The prior heat treatment of the samples led to the samples showing either a ferritic grain structure or a martensitic lath structure [25]. Sample exposure duration can be seen described within the reference, e.g. DNB-XXX-1000 has been exposed for 1000 h. HRA-8547-4580 has been added as a reference sample [2]. All samples were exposed at 600 °C.

**Fig. 1:**
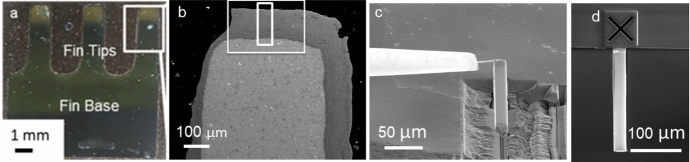
**a** and **b** showing the location the lift-out was prepared from, **c** the lift-out in progress, and **d** top-down view showing the lift-out connected to the Si holder

Samples were mounted in conductive Bakelite and polished to a 1 µm finish to ensure high quality scanning electron microscope (SEM) micrographs. The samples described here are all finned samples, an example of which is shown in Fig. 1a and b. All experimental work, unless indicated otherwise, was conducted across the top of the fin tips, due to the fin tips showing increased oxidation rates relative to the body and being more susceptible to breakaway oxidation [29].

SEM was performed using either a JOEL 7800F or 7100F field emission gun SEM (FEGSEM), both equipped with Oxford Instruments X-max 80 silicon drift energy dispersive x-ray spectroscopy (EDS) detectors. Typically, the backscatter electron (BSE) detector was chosen for SEM imaging with a beam accelerating voltage of 10 kV, a probe current of 12 nA and a working distance of 10 mm. For EDS analysis, typically 20 kV accelerating voltage was used with a probe current of between 14 and 16 nA and a working distance of 10 mm. Electron backscatter diffraction (EBSD) was performed in a JEOL 7100F SEM with an EDAX Hikari XP camera. This was performed at 20 kV with a beam current of 24 nA.

For 2D analysis of porosity, a series of BSE micrographs were taken through the oxide scale starting at the outer magnetite edge. These micrographs were then combined using ImageJ to create a montage which was thresholded to highlight the pores present. The porosity measurements were all validated manually to ensure that dark features were not included in the measurements (such as the carbon resin visible in Fig. 5a). The pore area fraction across the oxide was measured using ImageJ at each horizontal line of the montaged image. For additional details on this technique the reader is referred to the previous article [2].

Sample DNB-8401-3325 was selected for 3D analysis due to the shorter exposure time (3325 h) in comparison to HRA-8547-4580 in previous work [2] (4580 h) and because it is not described as having suffered breakaway oxidation. The centre of a fin was selected as the area of interest. The location of this is indicated in Fig. 1b.

For 3D analysis an FEI Helios G4 PFIB CXe was used to perform both the lift-out and successive cross-sectioning and imaging experiments. The large section was prepared at the fin tips of the sample with a target volume of 25 µm × 20 µm × 210 µm (Fig. 1c). A Pt layer of 120 µm x 20 µm x 5 µm was deposited to cover the total area of interest and account for material loss during milling. For the initial large lift-out, an ion beam current and voltage of 2.5 µA and 30 kV, respectively, were used. This allowed for rapid removal of material and the high current helped to ensure that no redeposition occurred. The lift-out was mounted on a Si holder, Fig. 1d.

For the successive cross-sectioning and imaging, milling of the sample was performed using an ion beam current of 15 nA at 30 kV, slices of 60 nm thickness were chosen, and a 5° rocking mill was used to decrease curtaining. These milling parameters were chosen as a compromise between milling speed and ion beam damage at the region of interest. Both BSE and secondary electron (SE) micrographs were collected after each cross-section slice at an electron beam current of 6.4 nA at 10 kV. The total distance through the oxide milled was 210 µm with a total of 3195 slices spanning through the total oxide scale.

On completion of the successive cross-sectioning and imaging, the micrographs were cropped to maximise the area characterised while keeping the area consistent for every slice. In total, 373 μm^2^ of material was analysed for each slice. A fast Fourier transform (FFT) filter was applied to all micrographs in ImageJ to remove curtaining. After that, the data set was thresholded and imported into Dragonfly version 2022.2.0.1367 for reconstruction and analysis. Manual thresholding was completed using ImageJ prior to importing the data allowing for more accurate identification of the pores within the substrate. The data set was split up and thresholded in smaller data sets so as to minimise any merging of the pores due to the thresholding. Thresholding was applied manually to the middle slice of the batch and then to the remainder of the batch to remove everything but the porosity. Several micrographs from each batch were checked manually to ensure that the batch thresholding was correctly applied.

## Results

### Substrate Microstructure

**Fig. 2:**
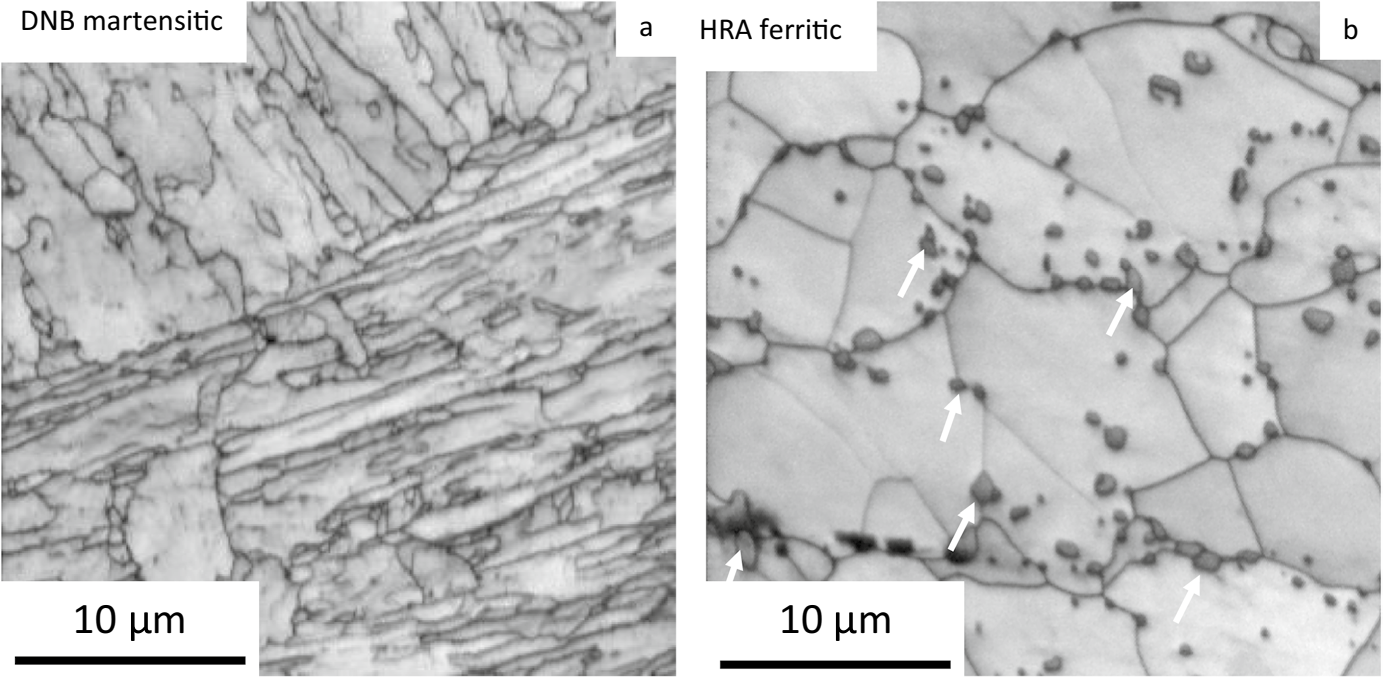
EBSD pattern quality maps showing the different microstructures of DNB **a** and HRA **b** material. The martensitic lath structure is clearly visible in the DNB material and the equiaxed grains can be seen in the HRA material. Carbide particles are highlighted with arrows in the ferritic material.

EBSD pattern quality maps of each of the unexposed substrate materials studied can be seen in Fig. 2. The DNB sample shows a martensitic lath structure, whereas the HRA shows an equiaxed ferritic structure. The lath structure can be clearly identified from the DNB martensitic material. Within the ferritic HRA sample, a selection of carbides can be seen highlighted with white arrows which are present along the grain boundaries and also within the grains. These carbides are less visible within the DNB material due to the difference in carbide size. Further discussion on the different microstructures and their effect on the oxidation properties of 9Cr-1Mo steel is discussed in previous work [25].

### SEM Analysis

**Fig. 3:**
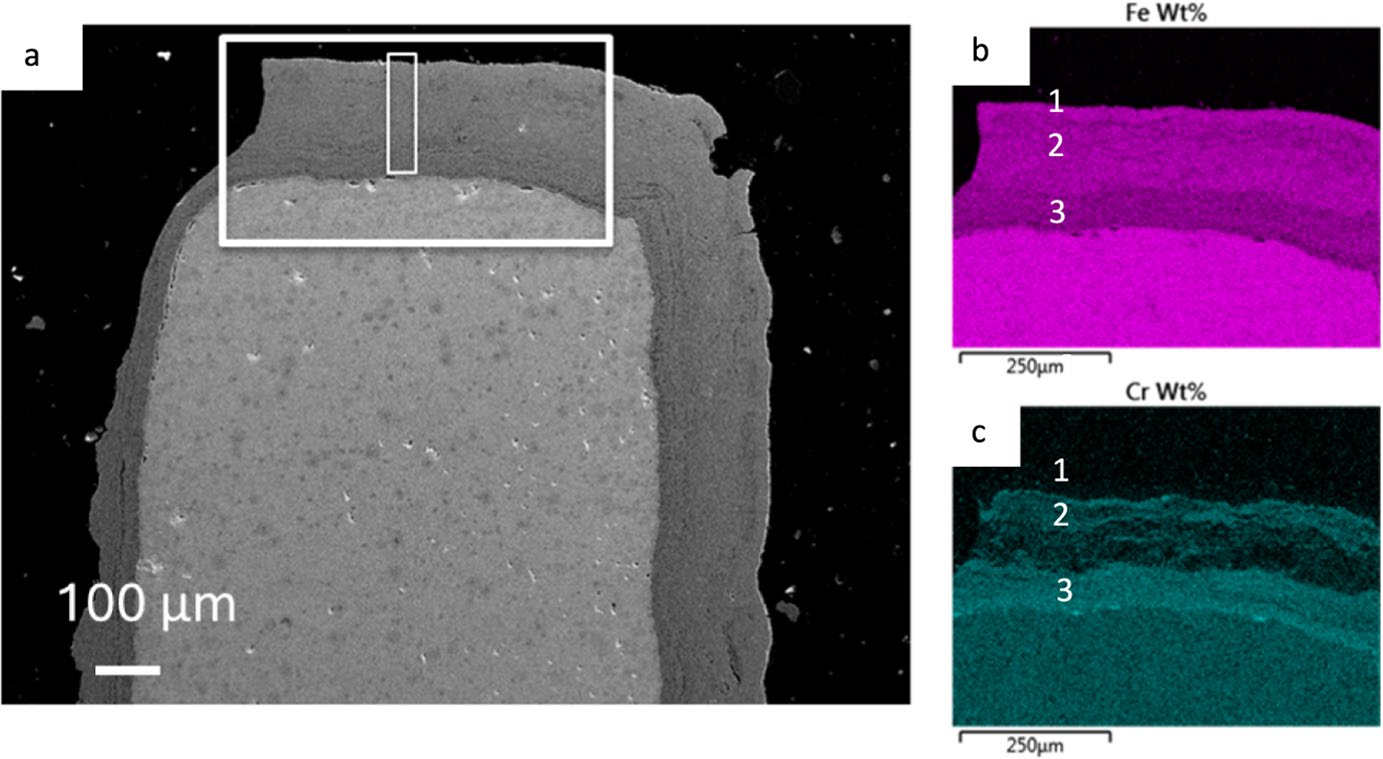
SEM micrograph showing the area where the large lift-out was prepared on sample DNB-8401–3325 with EDS maps of the oxide scale. **a** shows a low-magnification micrograph with the region where the EDS maps **b** and **c** were taken with a narrow box highlighting the region examined during the slice and view

Initial SEM analysis of the martensitic 9Cr-1Mo steel was performed at the area of interest towards the fin tips of the sample. Within the elemental maps shown in Fig. 3, there are three regions which are labelled. These areas show variations in the Fe and Cr concentrations through the oxide layer. The outermost Sect. 1 of the oxide is Fe-rich and shows no/little Cr presence. There is a middle Sect. 2 of the oxide which shows a mixture of both Fe and Cr. The innermost layer (3) shows a decrease in Fe relative to the other two outer layers, and an increase in Cr. The elemental concentrations seen in this layer are what would be expected of a typical Cr-rich spinel formed on 9Cr-1Mo steel exposed to CO_2_ [2, 8–10].

**Fig. 4:**
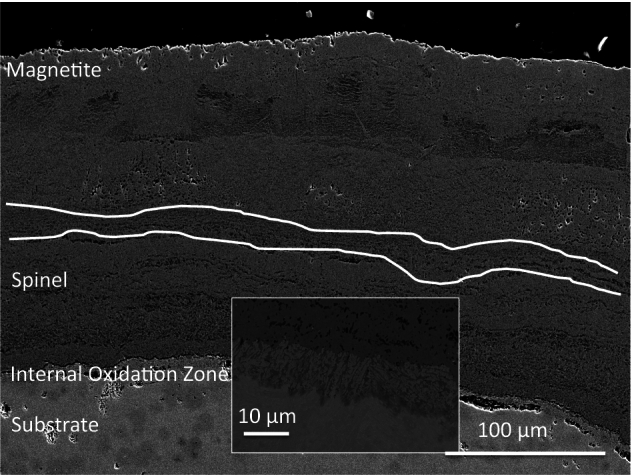
SEM micrograph from the fin tip of sample DNB-8305-3325 showing the oxide morphology present. The different oxide layers can be seen labelled. The insert shows a higher magnification micrograph of the Internal Oxidation Zone (IOZ)

An SEM micrograph of DNB-8305-3325 can be seen in Fig. 4 where several different layers can be seen. From the micrograph, the pores can be seen running parallel to the metal/oxide/air interfaces in a banded formation. An example can be seen highlighted in white in Fig. 3. This banding can be seen to be more pronounced towards the substrate, with the pores varying in size throughout the oxide. This is typical of 9Cr-1Mo steel exposed to CO_2_-rich atmospheres [2, 9]. The middle of the oxide appears more complex with both a non-porous and porous oxide forming.

**Fig. 5:**
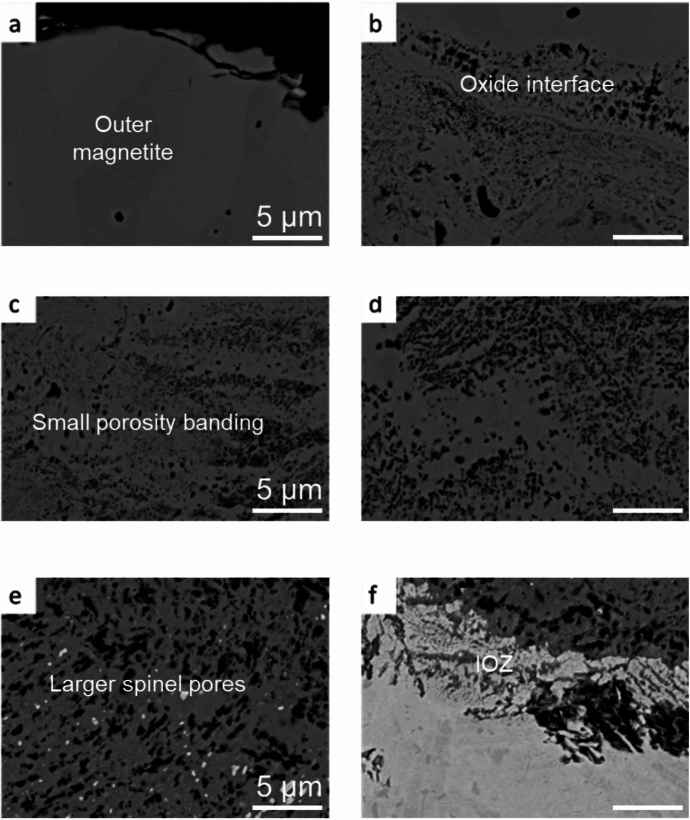
Micrographs showing the various porosity morphologies observed through the oxide scale formed on 9Cr-1Mo steel exposed to CO_2_

Through the oxide, there is a range of different types of porosity present. Micrographs taken at various points through the oxide scale can be seen in Fig. 5. The micrographs progress from (a) at the outer edge of the magnetite to (f) at the Internal Oxidation Zone (IOZ) between the substrate and the oxide. The various porosity morphologies seen can be described as;

**Position (a):** Small (> 1 μm) circular pores. From the SEM analysis these pores appear to show no interconnectivity with each other. They are observed across the magnetite oxide layer, seemingly at random.

**Position (b):** At the interface between the magnetite and the inner spinel oxide layer there is repeated banding of porosity, varying in size from nano-porosity to pores greater than 1 μm.

**Position (c):** Within the spinel layer the morphology varies. There are clusters of porosity present at some locations, whereas others show relatively random stand-alone pores, like those observed at position (a). The pores increase in size into the spinel oxide layer from the surface.

**Position (d):** Towards the middle of the spinel layer, the pores are larger, but are still present in clusters. There is banding of oxide showing little porosity, but there are some individual pores. The banding of the pores parallel to the oxide edge is still present, but the pores are larger in size.

**Position (e):** Towards the substrate, there is a mixture of both the porous oxide and the oxide showing little porosity. There are bright spots present in the oxide which have been previously identified as the remains of substrate carbides which have not oxidised as the substrate metal oxidises [2]. These are Fe-rich with a Cr shell which has oxidised and formed a protective Cr layer around the inner carbide [2].

**Position (f):** The internal Oxidation Zone (IOZ) forms at the interface between the substrate and the growing spinel oxide layer. It consists of Cr-rich oxide within the steel matrix. The penetrating oxide typically follows the lath/grain boundaries due to the Cr-enrichment from the substrate carbides [2, 25]. Pores can be seen in close proximity to the substrate highlighting that the porosity within the oxide forms during the initial stages of oxidation.

The oxide formed on the surface of martensitic DNB-8401-3325 is thicker in comparison to the oxide formed on the surface of the ferritic HRA-8547-4580 [2], with thicknesses of 210 and 120 μm after exposure for 3325 and 4580 h, respectively. This shows that the martensite substrate microstructure leads to the growth of a thicker oxide, even after a lower exposure time.

**Fig. 6:**
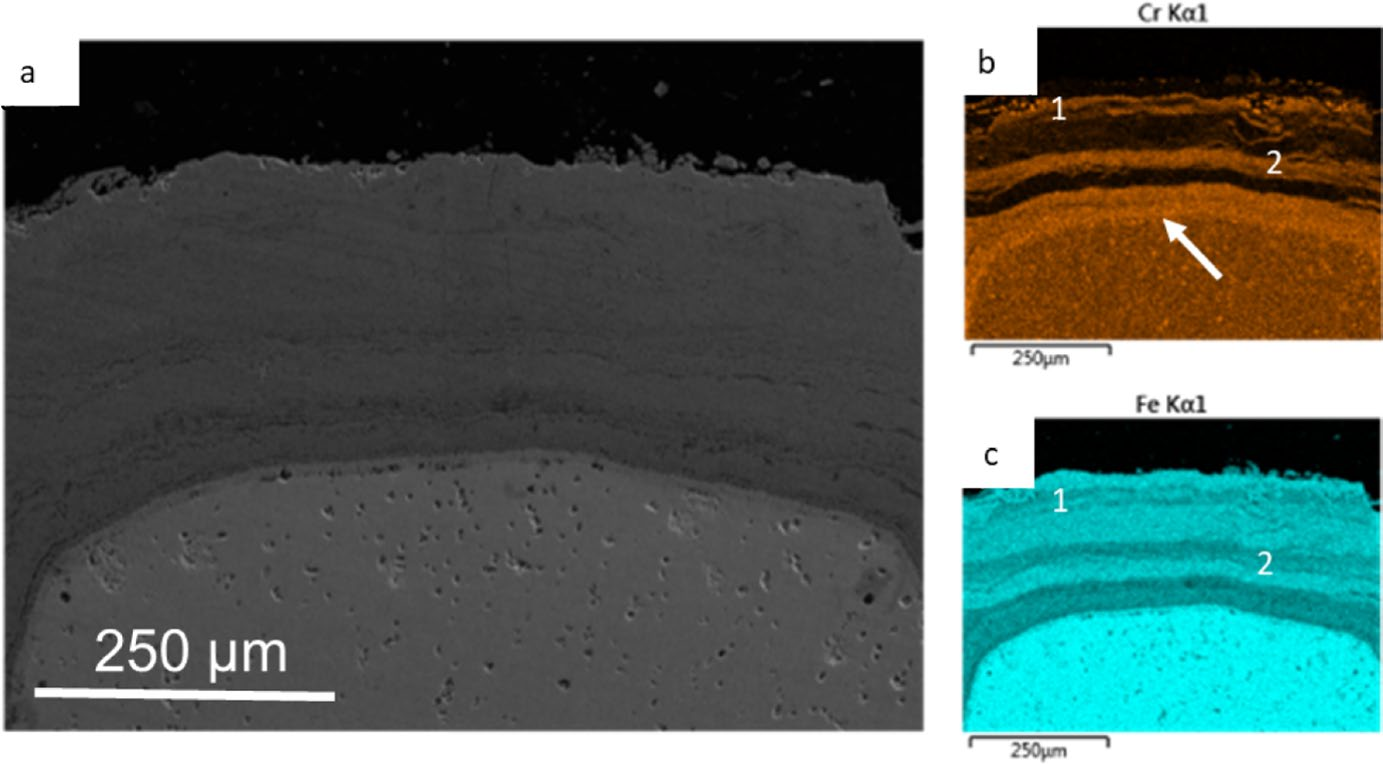
Micrograph with corresponding EDS maps showing the oxide formed on sample DNB-8401–5845. Repeating Cr- and Fe-rich bands can be seen through the total oxide scale with the “current” Cr-rich spinel at the interface labelled

The oxide layers are seen to repeat within the oxide scale at the tip location on this sample. This can be seen in Fig. 3 in the Cr map, but a more pronounced example can be seen in Fig. 6 with an EDS of sample DNB-8401-5845 (5845 h exposure). In comparison to DNB-8401-3325 this sample is described as having suffered breakaway oxidation. The distinct elemental layers can be seen. With evidence for two previous Cr-rich layers both highlighted with a 1 and 2 and the current protective Cr-rich oxide layer highlighted with a white arrow in the Cr map.

Layer 1 shows a Cr layer which appears to have dispersed into the surrounding Fe-rich regions, in comparison to layer 2. This layer can be identified from both the Cr and Fe maps. This would have been the initial Cr-rich spinel layer formed during early exposure of the material. In the Cr map, to the left of the (1) label and above the (2) label, variations within both the Fe and Cr maps can be seen.

From the Cr-rich layer at this location, it could be assumed that this region of the oxide shows the location of the prior substrate edge, which would agree with the location described by the available space model [8]. The layer labelled 2 shows a Cr-rich layer, typical of what would be expected from a duplex spinel oxide. These repeating layers have a different morphology and add complexity to the oxide formed on martensitic 9Cr-1Mo steel. This sample has been described as having suffered breakaway oxidation, and the formation of the repeating layers may be due to the steel reforming an inhibitive spinel once the spinel stops being protective.

These repeating layers will have influenced the oxide characteristics of the material and also led to more of the substrate being oxidised in comparison with the case in which only a duplex oxide had formed.

### 2D Porosity Area Fraction Measurements (SEM Micrographs)

**Fig. 7:**
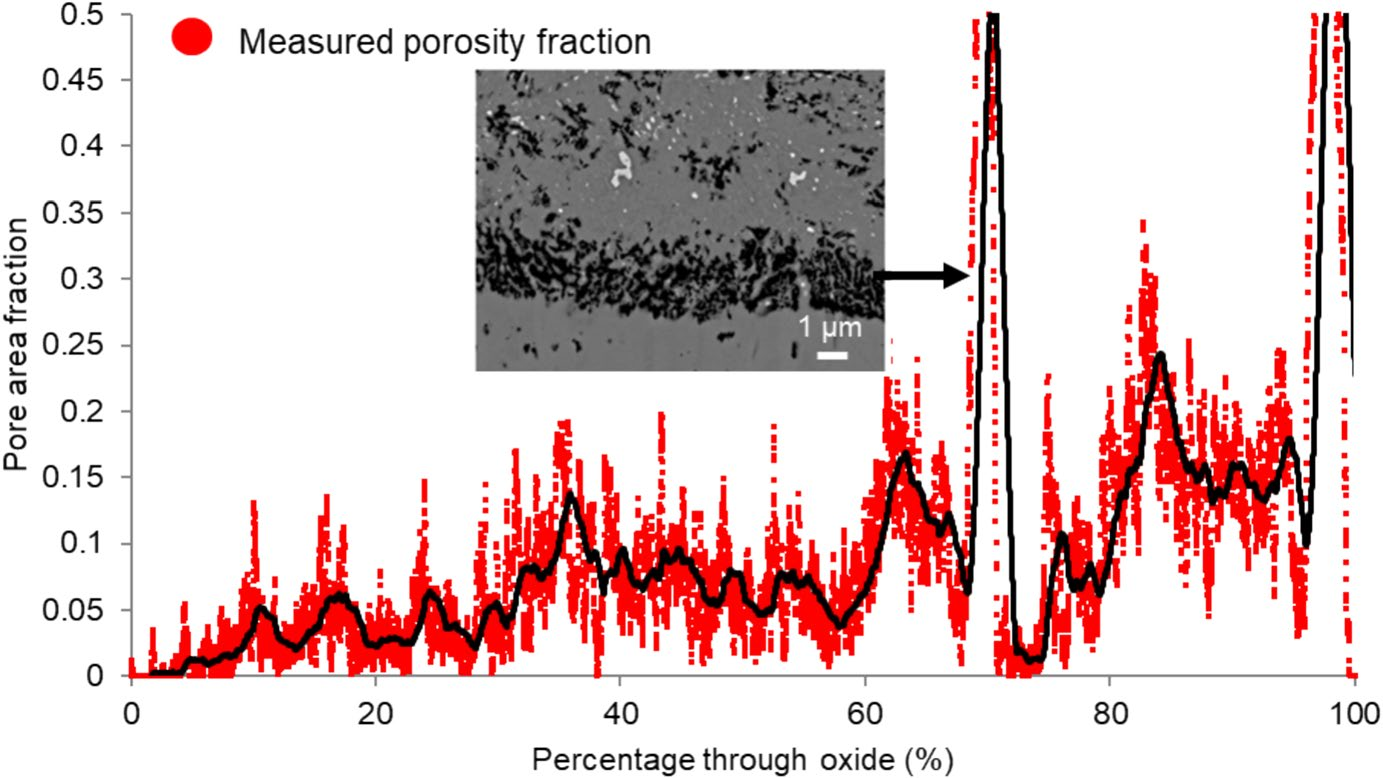
2D Porosity measurements taken through the oxide at the fin tip of sample HRA-8401-3325. Percentage through oxide indicates 0 at the outermost region of magnetite and 100% at the 9Cr substrate below where the slice and view were stopped. Black line indicates a moving average of 200 points. The insert shows a micrograph demonstrating how the peak porosity values are formed\\ within the scan

The porosity of the oxide was measured using SEM micrographs (Fig. 7), with a moving average trendline (200 points, black) applied. The max area fraction peaks of the porosity banding are seen at 70 and 95% through the oxide. These are due to the presence of large pore bands distorting the measured porosity in these regions. An example of such a band can be seen in the insert in Fig. 7. This localised banding is seen on a larger scale across the sample in Fig. 3 and can lead to high localised measurements recorded using the SEM-based 2D porosity measurement technique. These measurements are not fully representative of the total porosity within the scale as the pore structure is 3D, whereas this method only measures data in one plane (1D). For further details see the Experimental Section. For simple measurements, the pore fractions measured will be comparable; however, for complex structures this may not be representative of the actual behaviour due to the presence of large pores, voids, grain pull out.

### 3D Reconstruction of Porosity

**Fig. 8:**
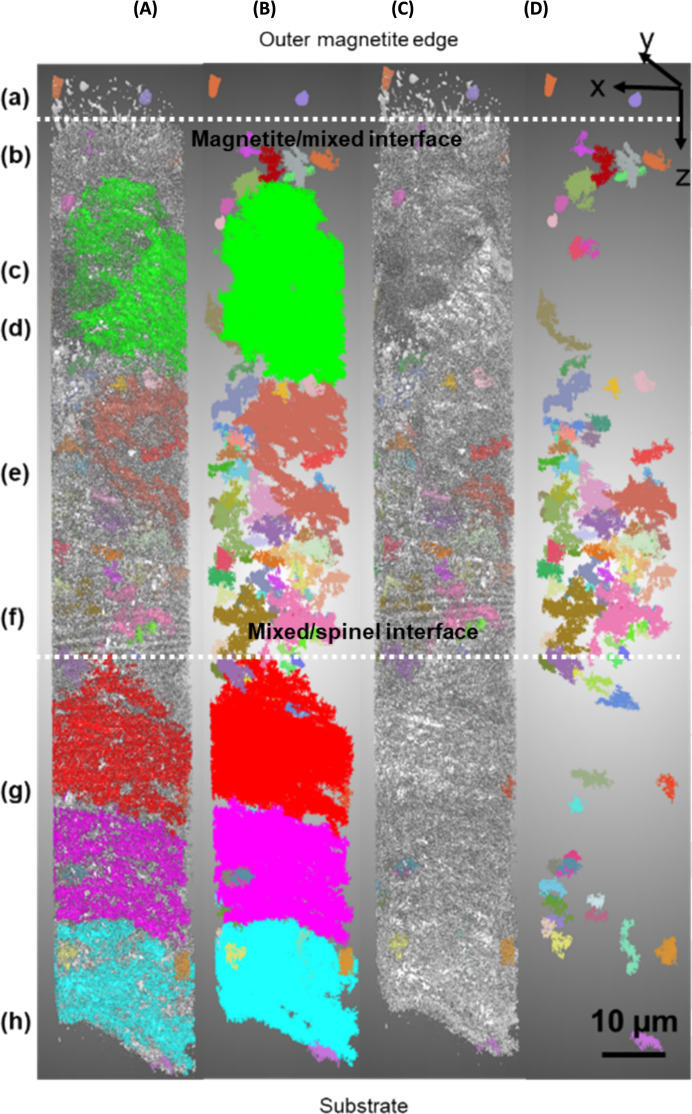
Reconstructions of the total 3D data set for sample DNB-8401-3325. **A** shows the total porosity with the largest 200 pores coloured. **B** shows the largest 200 pores with the remainder removed. **C** shows total porosity with the largest 200 pores coloured, but with the largest 5 pores removed. **D** shows 200 largest pores with the 5 largest pores removed. The Y axis letters refer to the different locations through the reconstruction which correspond to the micrographs shown in Fig. 9

A full 3D reconstruction through the total oxide scale of the porosity within the sample DNB-8401-3325 can be seen in Fig. 8. This Figure shows the reconstruction from top down (outer oxide edge at the top) and from both sides of the lift-out. Due to the complexity and high volume of porosity within the oxide, the largest 200 pores were coloured to highlight their distribution with the remainder of the porosity present in white. Each individual pore will be a single colour (although colours are repeated through the reconstruction). These colours are arbitrary and applied automatically by the software to give a good colour distribution through the reconstruction. This is shown in Fig. 8A. The porosity through the oxide showing only the largest 200 pores can be seen in Fig. 8B. The pore volume was calculated using Dragonfly software and the smaller regions of porosity could be removed from the reconstruction. The pores removed may have been larger in total volume due to interconnectivity outside of the selected area for analysis.

**Fig. 9:**
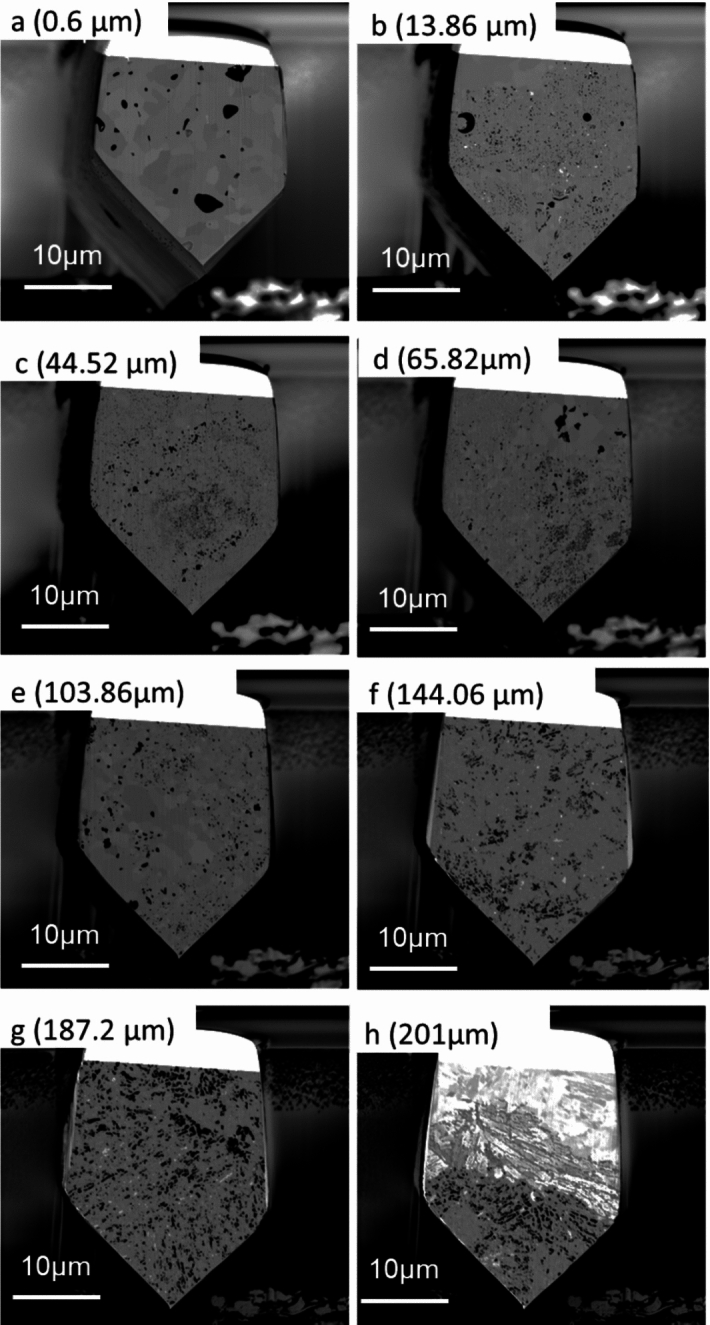
Micrographs showing various slices from locations through the slice and view. Letters shown correspond to the respective letters shown down the side of Fig. 8. Distance through the slice and view can be seen labelled on each respective micrograph

The five largest pores were removed from the reconstructions shown in Fig. 8A and B with the remaining pores shown in Fig. 8C and D. To the left of the Figure, letters (a) through (h) indicate the locations of the image slices shown in Fig. 9.

The magnetite layer on the outside of the oxide shows individual pores with little interconnectivity or connectivity with the next oxide layer.

At the interface between the magnetite and the inner oxide there are many pores present. From the cross-section, these pores appear relatively small, but in the reconstruction, these show interconnectivity. There is a cluster of smaller pores in this location; these are clearly highlighted in Fig. 8 (b&d) where this cluster of pores is present at the interface between the magnetite and inner oxide layer. A micrograph showing this region of porosity from the slice and view is shown in Fig. 9b where a high area frequency of porosity is observed.

Moving further into the oxide, a large pore can be seen in green. This large pore within the oxide spans ~ 30 µm microns and makes up the majority of the porosity in this region. One limitation of any reconstruction is the limited volume which can be reconstructed and analysed. Several pores within this reconstruction may be connected outside the selected region for reconstruction.

Moving further into the oxide, location (d, e, f), a high frequency of smaller pores is present, identifiable from the various colours present. There is a range of different morphologies present within this oxide layer, with little full interconnectivity between the pores in this location. The grains within the oxide vary between larger grains, more typical in size to magnetite (see Fig. 9a as a reference for magnetite grain type with Fig. 9d and e for the mixed oxide layer). This behaviour was observed in the SEM imaging (see Fig. 3), with both magnetite type and spinel type grains present but not fully identified as a mixed layer within the oxide. A summary of the various pore types is described below, linking the areas labelled within both Figs. 8 and 9. These are summarised as;

- The magnetite outer layer. This is characterised by larger individual grains within the layer.
- The magnetite mixed interface. The pore morphology observed in the inner layer is fine, as seen in ferritic 9Cr-1Mo steel [3].
- A range of pore sizes is observed within this region. There is a large pore spanning through this region, but the majority of the porosity is smaller localised individual pores. The larger grains show a different morphology in comparison to the smaller spinel grains.
- Similar to (c), the structure shows a mixture of larger grains and pores. Some larger grains are surrounded by smaller grains. Two distinct types of porosity are seen, with smaller fine pores linked with the smaller grains, and larger pores associated with the larger grains.
- This slice shows a high proportion of larger grains with more individual porosity (not interconnected). From the reconstruction this is the area where the majority of the porosity present is from smaller clusters of pores.
- This area shows a more typical spinel oxide appearance; the grain size increases in the slices, and the reconstruction shows an increase in pore interconnectivity from this location towards the substrate.
- Similar in appearance to location (f), but the pore size is larger in the slices. There are also brighter regions which have been previously shown to be carbides which have resisted oxidation at the oxidation front [30]
- IOZ pores and carbides are present within the newly formed oxide. The oxide is penetrating the substrate ahead of the oxidation front forming an IOZ. The IOZ can be seen to be following the lath boundaries within the substrate as previously shown [25]. Pores are present in the oxide close to the substrate highlighting how quickly they form after the oxide moves deeper into the substrate.

The oxide scale formed on martensitic 9Cr-1Mo materials shows a different morphology in comparison to the oxide which was previously seen on ferritic 9Cr-1Mo steel [2]. In the previous study, the oxide ratio was seen to be ~ 50:50 between magnetite and spinel layer which agrees with the available space model and dimensional metrology results [4, 8, 31, 32] whereas a similar ratio is not observed here. The ratio of magnetite to remaining oxide observed for this martensitic steel is ~ 10:90.

### Porosity Area Fraction Measurements (FIB/SEM Micrographs)

**Fig. 10:**
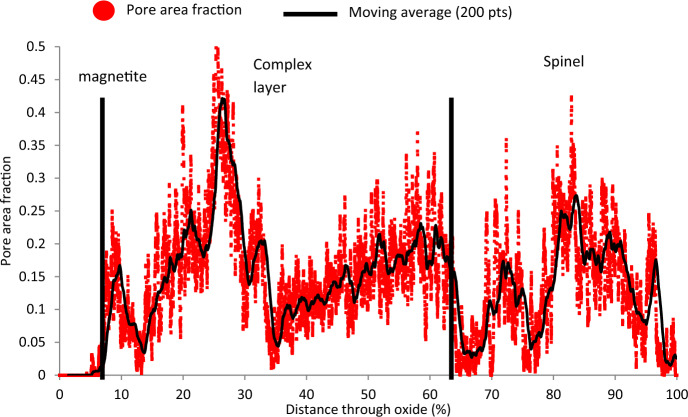
2D SEM Porosity measurements taken through the oxide at the fin tip of sample DNB-8401–3325. Percentage through oxide indicates 0 at the outermost region of magnetite and 100% at the 9Cr substrate below where the slice and view was stopped. The black line highlights a moving average of 200 points

The porosity of the through-thickness oxide was measured using SEM micrographs taken during the FIB slice and view (2D data from a 3D data set). For each slice, the area fraction was measured and then plotted as a function of distance through the oxide scale. The results can be seen in Fig. 10.

The outermost ~ 10% of the oxide shows little measured porosity. This region is the magnetite oxide layer. Through the rest of the oxide towards the substrate, the oxide porosity undulates which coincides with porosity banding seen through the oxide scale, shown previously in Figs. 3 and 5. In comparison to the ferritic steel’s porosity measurements performed using the same techniques [2], the agreement between the 2D SEM method and the 3D FIB method is not as consistent for the martensitic material. This is due to the increased complexity within the oxide formed on the martensitic steel relative to the ferritic steel leading to an inhomogeneous oxide where individual features can skew the measured porosity (as seen in Fig. 7). The total average porosity through the scale (measured from individual slices) is 10.7% with a median of 10.1% porosity observed within the oxide scale. And for the ferritic steel the total average porosity through the scale was 2.06% and the median porosity observed was 5.1%. This variation is due to the relatively large magnetite layer (50% of total oxide scale for ferritic steel) showing little porosity which lowers the total average measured.

**Fig. 11:**
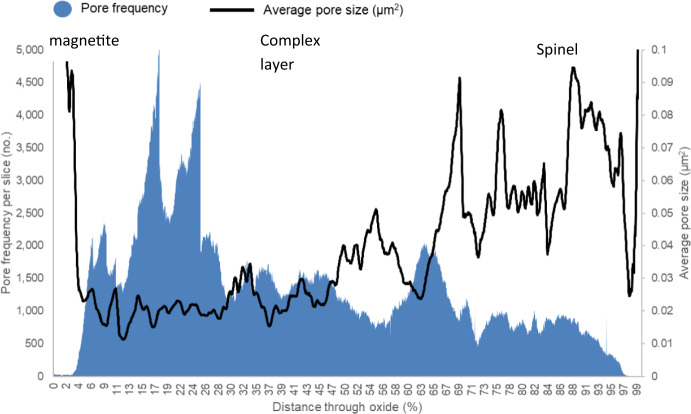
Chart showing the measured pore area frequency and average pore size for sample DNB-8401-3325. The pore frequency per slice with average 2D pore size can be seen through the oxide scale

The pore frequency and pore size were measured from each slice of the FIB-SEM micrographs, with the results shown in Fig. 11. The regions showing high frequency have smaller pores and the lower frequency showing larger pores. The outer magnetite region shows a high average pore size, but low area frequency, which can be seen from the SEM micrographs shown in Fig. 5. This was also seen within the magnetite layer of ferritic material with these larger voids/pores thought to be present due to growth defects during oxidation.

Within the complex mixed layer in Fig. 11, an increase in the pore area fraction can be seen which is linked with a decrease in pore size and frequency. This can also be seen from the SEM micrographs, Fig. 5b and c. In the complex layer, the average pore size increases with the area frequency decreasing. This can be seen in the reconstruction of porosity, Figs. 8 and 9, regions labelled (e) and (f) with several larger interconnected pores present within this area of the oxide.

**Fig. 12:**
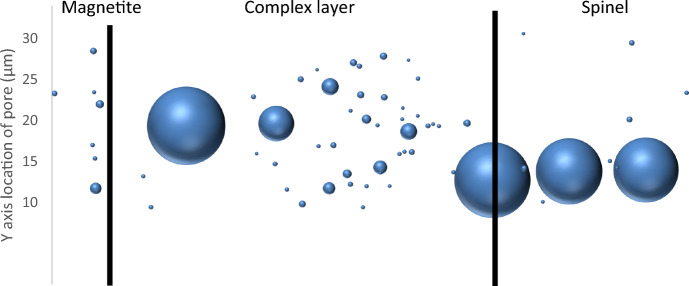
Chart showing the Y and Z locations of the 100 largest pores through the oxide scale with their relative size overlaid

Figure 12 shows the Y and Z locations of the 100 largest pores within the oxide scale, with their relative volumes represented by the “bubble” size. The centre of the bubble is positioned at the centre of the pore so it may not fully represent the overlaps taking place between the larger pores. The results highlight that the inner spinel layer consists of three main pores, whereas the middle mixed layer contains a larger frequency of smaller pores.

**Fig. 13:**
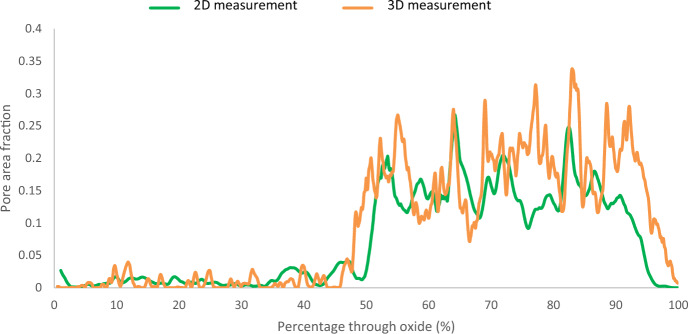
Comparison between 2D and 3D porosity measurements taken at the fin tip of a ferritic 9Cr-1Mo steel exposed to CO_2_ for 4580 h. This figure has been replotted from previous work by the authors [2]

The spinel oxide layer shows a decrease in the area fraction of pores, but an increase in the average pore size. This can be seen in Fig. 5d and e, with the pores present in this region being larger in comparison to those closer to the magnetite layer (Fig. 5b). The frequency of pores decreases towards the IOZ/substrate interface with a sharp increase in the average size at the interface. The measurements from the FIB-SEM method show different results in comparison to the SEM method. For the ferritic material [2], good agreement was achieved. The figure comparing the FIB-SEM and SEM methods is shown in Fig. 13. This is attributed to the increase in complexity in the oxide formed on the martensitic material in comparison to the oxide formed on the ferritic substrate resulting in more localised features which may give unrepresentative measurements of porosity when only considering one plane (see Fig. 7).

For the martensitic material, the main observed difference between the SEM technique and the FIB technique is the smoothing of high porosity regions where a localised pore feature misrepresents porosity at a certain region, for example pore banding (see Fig. 7). The FIB measurements show regions of increased porosity, followed by decreases throughout the oxide which are a result of the porosity banding within the oxide, as seen in the surface micrographs (Figs. 3, 4, and 5). From the porosity volume fraction measurements (FIB-SEM imaging), it is possible to identify three main regions, the outer magnetite, the inner complex layer and the Cr-rich spinel layer. These can be seen labelled on Fig. 11.

To further characterise this porosity, reconstructions were completed of sections within the oxide, with all the pores included in the analysis. These are shown in Fig. 15 through Fig. 18.

**Fig. 14:**
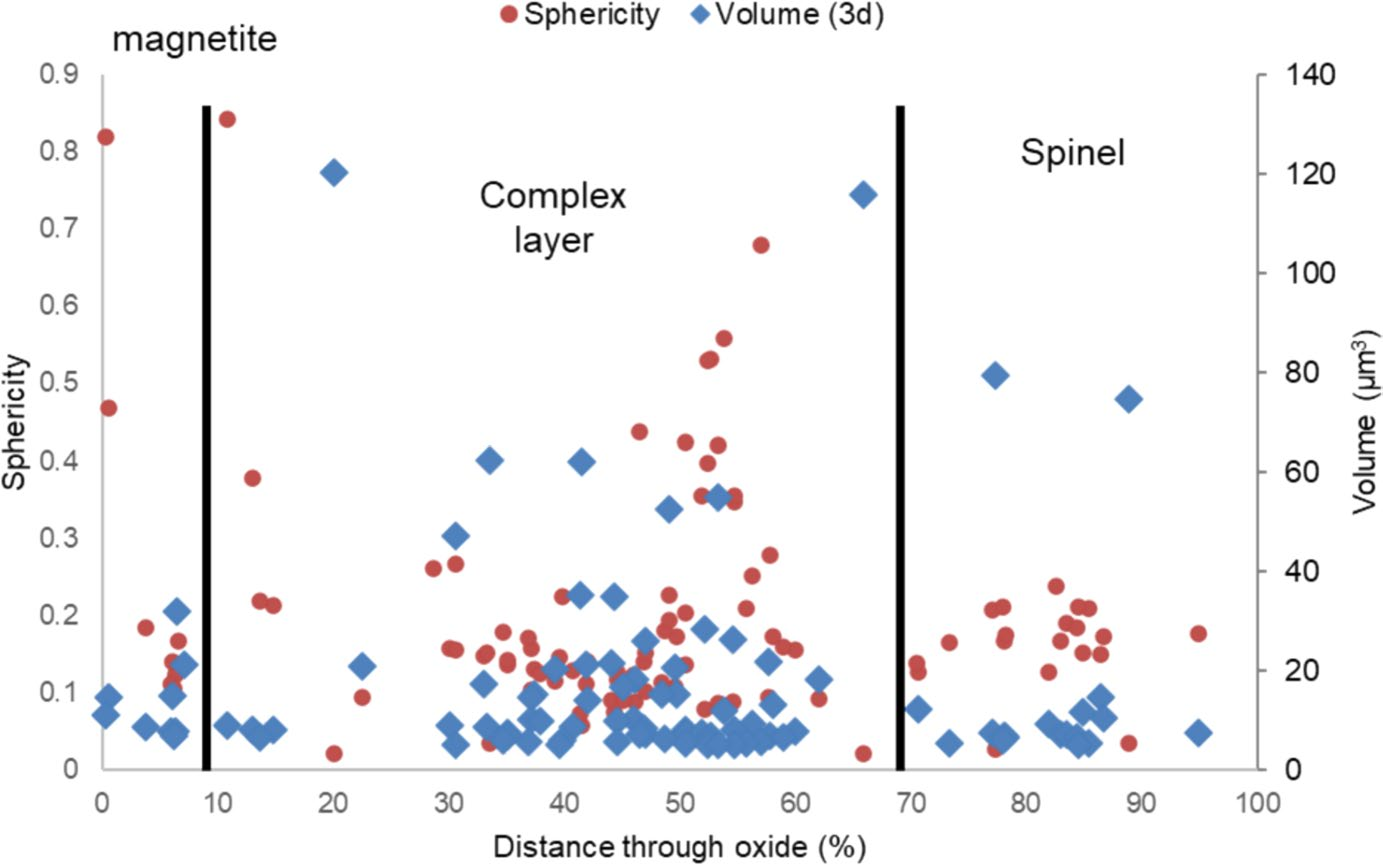
Sphericity and volume plotted against distance through oxide for 100 pores with the top 5 largest excluded

Sphericity of the pores (3D) can be extracted from the 3D data set, with a measurement of 1 meaning a perfect sphere. The sphericity of the 100 largest pores was plotted as a function of distance through the oxide with the pore volume. This can be seen in Fig. 14 with the three main oxide areas labelled as in previous figures. The four largest pores were removed due to these showing a far greater volume in comparison to the next 100 as seen in Fig. 8

From both the sphericity and the volume measurements through the oxide, the different layers can be identified. The outer magnetite layer shows a large range of both spherical and non-spherical pores. The spinel layer shows consistent pore volume and sphericity throughout towards the substrate and the complex layer in the middle shows a combination of this with some regions showing higher sphericity and some showing lower sphericity.

The mixed layer shows a combination of both less-spherical and more spherical porosity with there being an increase in the sphericity towards the interface with the spinel boundary where the sphericity decreases.

### Magnetite Interface (0–60 µm from the Oxide Air Interface, 0–28%)

**Fig. 15:**
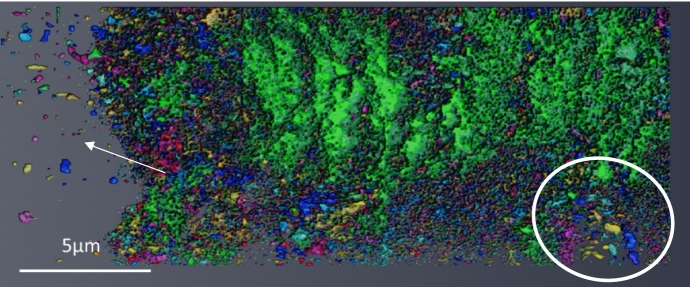
Section one of the porosity reconstruction of sample DNB-8418-3325, micrograph taken in the ZY axis, with the magnetite to the left

The first segment of reconstruction (~ 60 µm) shows the interface between the outermost magnetite and the inner spinel/complex oxide layer and can be seen in Fig. 15. The magnetite layer shows some spherical porosity with little interconnectivity. At the initial interface between the magnetite and the inner oxide layer, a high frequency of smaller pores can be seen. This banding of porosity is also observed in the SEM analysis shown in Fig. 5b and c with fine porosity banding occurring at this interface. This band of fine pores was also observed at the interface between magnetite layer and spinel in the ferritic sample [2]. This thin outer magnetite layer has been previously reported within 9Cr-1Mo steels exposed to CO_2_ which found that at later stages of exposure, the magnetite growth rate slows in comparison to the inner oxide layer which grows more rapidly [8].

A large pore can be seen spanning the remainder of this section with several smaller pores around the edges of the lift-out volume. The circle highlights an area showing a region where there is a cluster of smaller pores, similar in size and morphology to those seen in the outer magnetite layer (indicated by an arrow) demonstrating the complexity of oxide formation.

### Mixed Complex Layer (60–120 µm 28–56%)

**Fig. 16:**
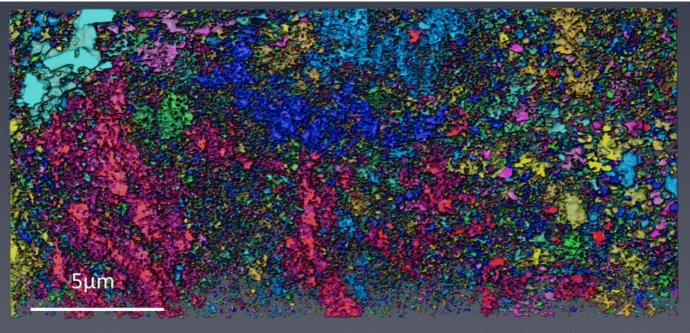
3D reconstruction of the porosity from section two (slice 1001–2000). This area shows a high frequency of porosity with both magnetite-type grains and spinel grains present

The second section reconstructed is the mixed complex layer, Fig. 16. As seen from the complete reconstruction (Fig. 8, area d, e) this area shows little interconnectivity through the oxide in comparison to the outer section and the region closest to the magnetite. Across this region there are also multiple pore morphologies with both spherical individual pores present and smaller regions showing some interconnectivity but spanning a relatively small area. In Fig. 9e, a white box can be seen highlighting where larger individual magnetite-type grains can be seen with smaller spinel-type grains surrounding them.

### Spinel Oxide Layer (120–180 µm 56–84%)

**Fig. 17:**
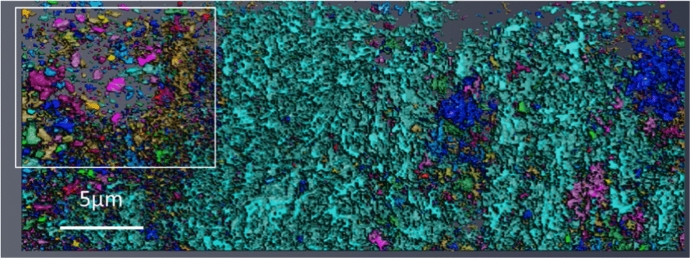
3D reconstruction of section three, slices 2001–2800. This section spans from the mixed layer into the spinel layer

The 120–180 µm reconstruction can be seen in Fig. 17. A large area of porosity can be seen as a single pore (indicated as a single colour) and spans a large region of this section. The banding of larger pores can be clearly identified and matches the morphology seen from the SEM micrographs in Fig. 5 c and d and the complete reconstruction in Fig. 8f through h.

The region highlighted with a white box shows a cluster of individual more spherical pores similar in morphology to those seen in the outer magnetite layer. A cross-section micrograph highlighting a similar region of porosity can be seen in Fig. 9d.

This section of the porosity reconstruction is the region where the oxide changes from a mixed oxide, with the larger magnetite type grains and smaller spinel grains, to consist of only the smaller spinel oxide grains. This can be seen in the porosity morphology with the lack of the smaller individual pores present which are associated with the larger magnetite type grains.

### Spinel Layer to Substrate (180 µm to Substrate, 85–100%)

**Fig. 18:**
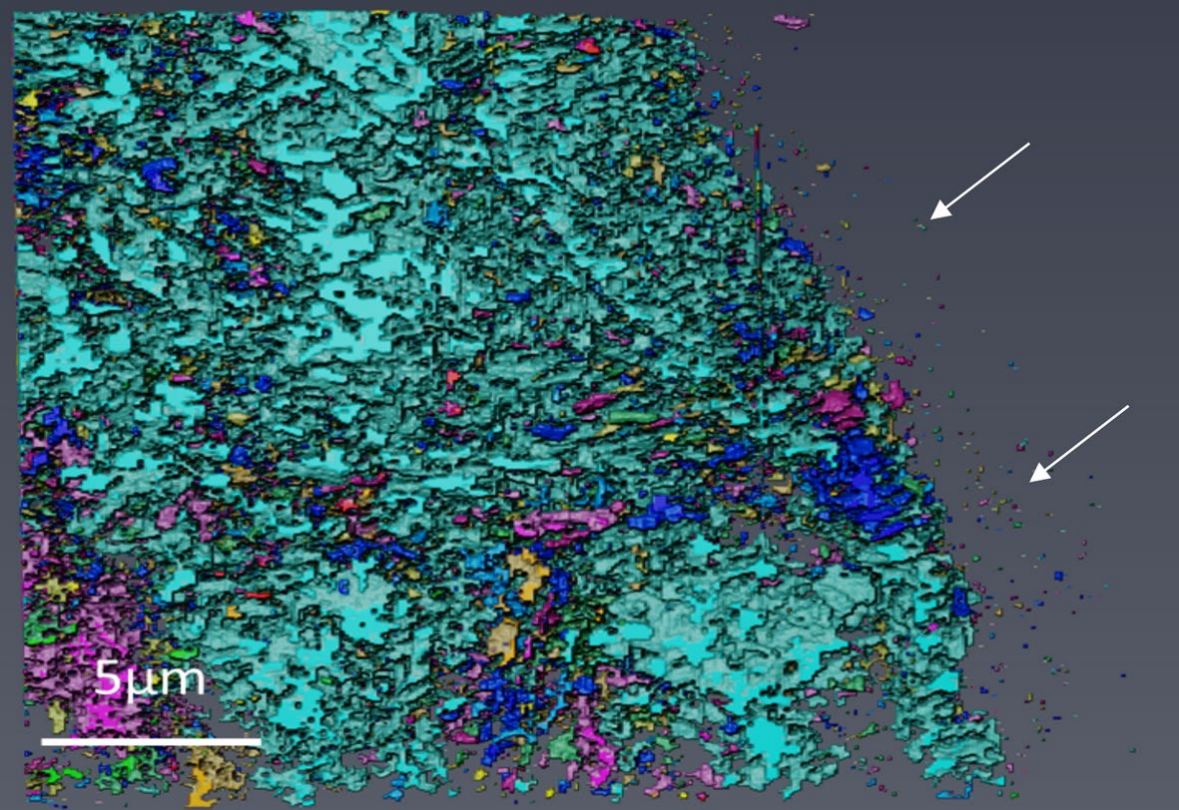
3D reconstruction of section four, slices 2800-substrate. The interface area is highlighted with white arrows

The final section of reconstruction shows the interface between the oxide and the substrate (Fig. 18). The large, interconnected pores are present throughout this region, including at the interface with the Internal Oxidation Zone (IOZ). Smaller pores can be seen within the IOZ with some examples highlighted with white arrows. These pores form on the oxidation of the substrate along the lath boundaries. For a cross-section view of this region of the sample, see Fig. 9h where the porosity can be seen within the IOZ.

Due to the sectioning of the data for the higher-resolution reconstructions, the pore distribution appears different in Fig. 15 through Fig. 18 in comparison to the complete reconstruction shown in Fig. 8, where the three large pores within the inner oxide layer can be clearly identified.

## Discussion

### Techniques

The work presented here has used a range of techniques to investigate the porosity through the oxide scale. The initial SEM method effectively gives a 1D porosity measurement through the oxide from a 2D data set. The FIB-SEM micrograph measurements provide a 2D porosity measurement through the oxide from a 3D dataset and the 3D reconstruction of the slices gives a true 3D analysis of the data.

In comparison to previous work, the more complex microstructure within the oxide formed on martensitic 9Cr-Mo steel in comparison to ferritic 9Cr-1Mo steel requires the use of 3D analysis to fully understand the microstructure and the porosity through the oxide scale. It has highlighted the presence of magnetite-type grains within what would have previously been considered the spinel layer. We can see the pore interconnectivity within each layer and how the pore type varies, whereas with typical SEM this is not possible.

This work has characterised the porosity in a relatively small region of the oxide, towards the fin tips which show a greater level of oxidation in comparison to the sides of the fins and also the dips between the two fins [8]. With any technique, the area selected is a compromise between experiment duration and cost, analysis time and resolution. It is expected that the region selected is representative of the oxide formed, although it may not fully highlight the interconnectivity of the porosity within the scale, because the pores present in this reconstruction may actually be interconnected outside the area of interest. The region characterised in this study was very heterogeneous, and from the lower magnification characterisation (low magnification micrographs and EDS maps) it is thought that although the morphology will vary throughout the oxide on the microscale the general structure will remain consistent.

### Porosity

In comparison to the previous 3D analysis of the oxide formed on a ferritic, equiaxed-grained material [2], the martensitic steel shows a far more complex oxide structure with a higher volume of porosity present showing greater interconnectivity (at certain regions). This can be identified from comparing the spinel layer of the previous ferritic steel and the spinel layer of the martensitic steel shown in Fig. 8. The oxide thickness of the martensitic steel is far greater than on the ferritic, but three large pores with a small amount of overlap span the total inner spinel layer. In the mixed layer, there is a far larger frequency of smaller pores which cannot be directly compared to the ferritic steel and the magnetite shows very little interconnectivity in both.

For the martensitic material studied here, the oxide forms repeating Cr/Fe-rich layers, which have not been previously observed in the oxide formed on the ferritic 9Cr-1Mo steels [2, 25, 30, 33]. These repeating layers are similar in composition to a typical spinel (Cr-rich) and magnetite (Fe-rich).

The repeated oxide layers alter the porosity of the oxide due to the magnetite showing a different porosity profile to the spinel and complex mixed layers leading to banding within these layers and an undulating porosity profile through the oxide.

The spinel oxide region towards the substrate shows a high level of pore interconnectivity, with three large pores spanning the total distance across the region sampled, with approximately 20% porosity volume fraction measured through this area. These pores may be connected outside the area characterised, and if so, would indicate the bulk of the inner spinel oxide layer shows interconnectivity across it.

The individual measured pore size (2D image per slice) increases towards the IOZ (See Fig. 11). The pore size increase is linked to the elemental distribution within the substrate, which in the later stages of oxidation leads to Cr enrichment towards grain boundaries due to carburisation [30]**.** This distribution affects the ability of the pores to form and coalesce during oxidation and leads to the variation in pore size within the spinel observed from near the magnetite layer to deep within the spinel [2, 28]**.**

The reconstruction also shows that in the volume investigated at the fin tip, there is no pore network spanning from the substrate to the outer edge of the oxide. The presence of such a network would allow for rapid inward diffusion of CO_2_ to the substrate, and subsequently a dramatic increase in the rate of oxidation, or breakaway. In comparison to the previous work investigating the porosity in the oxide formed in an equiaxed ferritic 9Cr-1Mo steel substrate [2], there is a far greater degree of interconnectivity shown in the current study. This greater degree of porosity and interconnectivity within the system will increase the surface area of the oxide, which may lead to an increase in the rate of the Boudouard reaction and could lead to a higher rate of carburisation [11, 14, 22–24], although this measurement was not performed here so a comparison cannot be made directly. This large volume of interconnected pores through the oxide scale may be one factor leading to an increased rate of oxidation in this material relative to ferritic material.

Analysis using PFIB has also allowed for greater area analysis through the oxide, with an area of ~ 30 µm × ~ 35 µm being characterized at every 60 nm through the thickness of the oxide. This has allowed the complex mixed oxide layer to be better characterized with both the larger grains and smaller spinel grains present in the same area, which, from SEM analysis, cannot be clearly identified. This area shows a high frequency of smaller interconnected pores; see Fig. 8D, locations (e) and (f). These pores are inhibited from further growth due to the presence of the larger magnetite grains.

### Oxide Layering

Due to the different oxide layers formed, the diffusion taking place is not as simple as in a typical equiaxed grained ferritic steel, where the magnetite grows outwards at a ~ 50:50 ratio to the inwards growing spinel layer, described by the available space model [5–11]. Within the mixed oxide layer on the martensitic substrate, there is banding of both magnetite grains and spinel oxide suggesting that the interface between spinel/mixed and magnetite may not be the original substrate interface. The continued growth of the oxide may therefore be taking place within the mixed oxide layer, as the oxide thickness continues to increase with exposure time, but the pure magnetite layer remains relatively consistent with exposure time, ~ 18 µm vs 22 µm (see DNB-8401-3325 (3325 h exposure) compared with DNB-8418-5845 (5845 h exposure), Figs. 3 and 4 with Fig. 6).

Multiple Cr-rich layers have been identified within one oxide, shown in Fig. 6, which may be due to the failure to form a fully protective scale upon initial oxidation and then reform a protective scale below, due to defects present on the surface before exposure or even the failure of the initial protective scale and the substrate has subsequently formed a Cr-rich inner protective Cr scale.

**Fig. 19:**
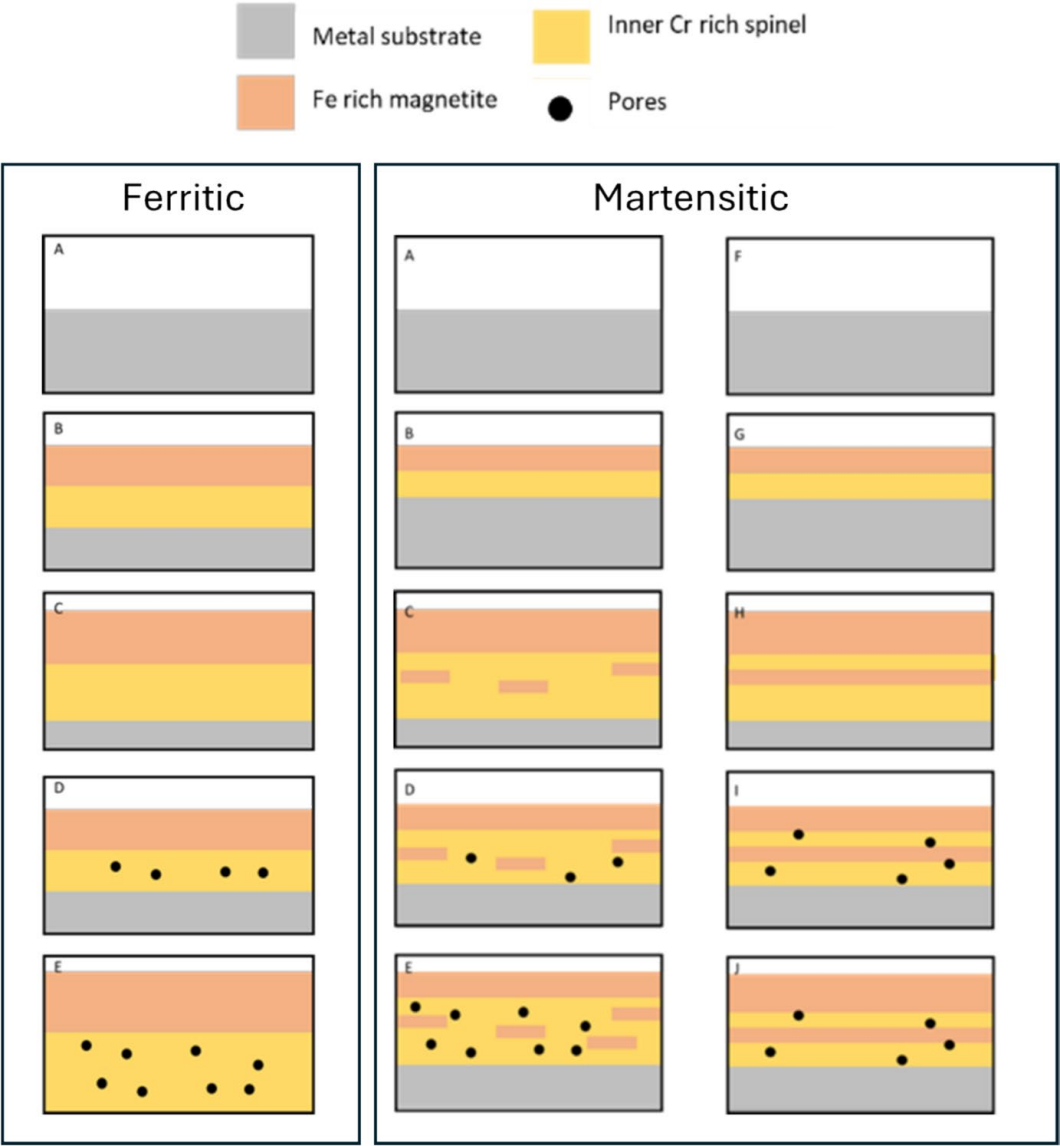
Schematic detailing the growth mechanisms for ferritic and martensitic 9Cr-1Mo steel exposed to a CO_2_. For the ferritic schematic, **a** initial outer metal interface, **b**, **c** oxide growth following the available space model, **d** pores develop in the spinel oxide layer and **e** the ratio between oxide layers deviates from the 50/50 ratio described by the available space model due to porosity present in the oxide. For the martensitic schematic: **a**, **f** initial outer metal interface, **b**, **c**, **g**, **h** oxide growth following the available space model, **d**, **i** pores develop in the spinel oxide layer and **e**, **j**

The schematics shown in Fig. 19 describe the oxidation process suggested to be taking place when the growth of a scale follows the available space model, as seen for equiaxed ferritic 9Cr-1Mo steel [2, 31]. The development of porosity is simplified and shown in 2D relative to the oxidation which is taking place in 3D and leads to the formation of more complex oxide structures. Figure 19d (ferritic) shows the development of pores within the oxide layer. This leads to the deviation from the 50:50 ratio, with a thicker spinel developing. This continues to develop unless the oxidation kinetics change, for example, due to breakaway initiation. Additional results discussing the porosity development of ferritic 9Cr-1Mo steel are discussed in previous work [2].

Figure 19 (martensitic) shows the proposed development of the oxide on the martensitic steel substrate exposed to a CO_2_-rich atmosphere. There are two paths of oxide development shown with one developing a multi-layer oxide with a combination of both magnetite type grains and spinel type in the same space (a through e), and one showing the formation of banding between the magnetite and spinel oxide layers (f through j). Initially, the oxide develops as seen on the ferritic substrate (Fig. 19 ferritic) with the Fe-rich magnetite oxide layer growing out from the initial substrate interface. The oxide initially grows following the available space model, forming the expected duplex oxide scale, with an Fe-rich magnetite oxide layer.

The Cr-rich spinel formed during initial exposure does not inhibit the corrosion effectively which leads to the inward diffusion of gas, causing the formation of the mixed oxide layer. Both the magnetite grains and spinel oxide grains grow at the various locations within the oxide, below the initial oxide formed, effectively following a localised available space model. This leads to the formation of the mixed oxide layer, characterised by the presence of both larger magnetite grains and spinel oxide in close proximity. The remains of the Cr-rich oxide layer formed during the initial oxidation can be seen in the EDS maps (Fig. 4) where there is the presence of both Fe and Cr. This mixed layer oxide formation can take place either locally or across the scale, producing a localised mixed oxide, or a multi-layer magnetite/spinel oxide layer (see Figs. 3 and 6). The outermost magnetite layer does not continue to grow once the mixed oxide layer has begun to form below, leading to the thin magnetite oxide layer, far thinner than that formed on ferritic 9Cr-1Mo exposed to CO_2_ [2, 8].

The mixed layers of alternating oxide layers lead to the formation of the parallel banding of porosity associated with 9Cr-1Mo exposed to CO_2_, with the pores inhibited from coalescing further into a large network due to the alternating oxide layers [27]. This can be seen in Figs. 4 and 5. This complex mixed layer offers less protection to the sample in comparison to a spinel layer and only offers limited oxidation resistance. The gas products from the Boudoard reaction can diffuse inwards to this region where they react with the outward diffusing Fe, as described by the available space model, but due to the presence of the spinel oxide, the mixed layer is formed with the outer oxide layer remaining.

This mechanism can lead to the formation of repeating layers, and may result in multi-layered oxide (e.g. two complete duplex oxide scales) being present, as shown in Fig. 6. It is thought that the oxide growth takes place in this way because of the prior martensitic lath structure of the substrate leading to an increased rate of oxidation in comparison to a ferritic microstructure. This results in the formation of an initial non-protective (or less protective) oxide [25, 30, 33]. Eventually this could lead to an enhanced rate of oxidation which would lead to the failure of the oxide scale and potentially subsequent spallation.

## Conclusions

The oxide scale formed on a martensitic 9Cr-1Mo steel has been characterised using a PFIB which has allowed for the analysis of a large volume of oxide in 3D. The porosity throughout the oxide has been characterised and the differences which arise due to a martensitic microstructure (relative to ferritic 9Cr-1Mo steel [2]) have been discussed.

From the experimental results shown, the following conclusions can be drawn: 

Comparison of techniques:

- For the more complex oxide structure seen here, the 2D SEM measurement technique is not as representative of the porosity due to the presence of localised pore features in the oxide. The 3D FIB method gives a more representative result.
- The 3D imaging technique is able to measure the porosity volume fraction through the oxide and to give size and volume data of the pores within the oxide.

Comparison between materials:

- The martensitic substrate microstructure leads to a more complex oxide than the ferritic steels which form the typical duplex oxide scale. This can be seen from the presence of the mixed oxide layer which is not observed in ferritic 9Cr-1Mo steel exposed to a CO_2_-rich atmosphere.
  - The total oxide scale shows a far greater level of porosity in comparison to the ferritic material.
  - The 3D reconstructions show a greater interconnectivity between the pores through the oxide, with three large pores spanning a large fraction of the total oxide.
  - The magnetite outer oxide layer is a smaller fraction of the total oxide than the ferritic substrate.
  - The total oxide is thicker (for similar exposure durations) and the total volume of porosity through the oxide is greater for martensitic steels.
- Three clear regions of pore and oxide morphology are observed within the total oxide;
  - Magnetite with infrequent non-interconnected pores showing a typical magnetite oxide appearance.
  - A complex oxide layer of larger magnetite grains and smaller spinel grains [2]. This region (~ 20% from outer edge to 60% towards substrate) can also show repeated Cr-rich oxide layers which suggest that these larger grains may be magnetite within the complex mixed layer.
  - A spinel layer where the pore interconnectivity is high and there are multiple large pores which span the total distance through the area studied. These large, interconnected pores give rise to the banding observed and are thought to take this form due to the elemental segregation during substrate carburisation.
- A mechanism has been proposed for the formation and development of the mixed oxide layer within the oxide, which describes how the magnetite layer at the outermost section of the oxide ceases to develop while the inner oxide continues to grow and develop with exposure time.

## Data Availability

No datasets were generated or analysed during the current study.
